# Suppression effect of body weight on the association between cigarette smoking and telomere length: the Bogalusa Heart Study

**DOI:** 10.18632/aging.102439

**Published:** 2019-11-09

**Authors:** Miaoying Yun, Shengxu Li, Yinkun Yan, Tao Zhang, Lydia Bazzano, Jiang He, Wei Chen

**Affiliations:** 1 Center on Translational Neuroscience, College of Life and Environment Sciences, Minzu University of China, Beijing 100081, China; 2 Children’s Minnesota Research Institute, Children’s Hospitals and Clinics of Minnesota, Minneapolis, MN 55404, USA; 3 Department of Epidemiology, School of Public Health and Tropical Medicine, Tulane University, New Orleans, LA 70112, USA; 4 Beijing Children’s Hospital, Capital Medical University, National Center for Children’s Health, Beijing 100045, China; 5 Department of Biostatistics, School of Public Health, Shandong University, Jinan, Shandong 250012, China

**Keywords:** telomere length, smoking, body mass index, suppression effect

## Abstract

This study aimed to dissect the direct effect of smoking and its indirect effect through body mass index (BMI) on leukocyte telomere length (LTL) and to distinguish the mediation and suppression effects of BMI. The study cohort included 1,037 adults (729 Whites and 308 African Americans; 42.1% males; mean age: 40.3 years) with LTL measurements by Southern blotting. General third variable models were used to distinguish the mediation and suppression effects of BMI on the smoking-LTL association. After adjusting for age, race, sex and alcohol drinking, the total effect of smoking on LTL was significant (standardized regression coefficient, β= -0.061, p=0.034) without BMI included in the model. With additional adjustment for BMI, the indirect effect of smoking on LTL through BMI was estimated at β= 0.011 (p=0.023), and the direct effect of smoking on LTL was strengthened to β= -0.072 (p=0.012). The results were similar when pack-years of smoking was used. The effect parameters did not differ significantly between race and sex groups. These results suggest that BMI has a suppression effect, not a mediation effect, on the smoking-LTL association, which potentially contributes to previous inconsistencies in the effect of smoking on LTL.

## INTRODUCTION

Telomeres, the TTAGGG tandem repeats at the ends of chromosomes that protect the end of chromosomes, become progressively shortened with each cell division [1]. As such, telomere length has been considered as a marker of biological aging, particularly cardiovascular aging [2, 3]. Extensive evidence has shown that age-dependent leukocyte telomere length (LTL) attrition is associated with cardiovascular risk factors such as race, sex, obesity, and unhealthy lifestyles, including excessive alcohol consumption, lack of physical activity and cigarette smoking [3–7].

Effects of cigarette smoking on inflammation and oxidative stress in humans, animals, and in vitro models are well documented [8, 9]. Exposure to harmful chemicals in cigarettes induces irreparable damage to the telomeric DNA through the mechanisms of increased inflammation and oxidative stress [10–12]. Despite the biological link, the literature is considerably inconsistent regarding the association between smoking and telomere length; some studies showed shorter telomeres with smoking, whereas a lack of a significant association was reported in the majority of studies [13]. Among various reasons, the role of a third variable is a potential explanation for these inconsistencies.

Systematic reviews showed a trend toward an inverse association of obesity and body mass index (BMI) with telomere length [14–16]. Furthermore, BMI is inversely associated with cigarette smoking [17, 18]. Obesity as a third external variable may have a mediation, confounding or suppression effect in the smoking-telomere length association studies [19]. To date, the nature of the complex relationship between BMI, cigarette smoking and LTL, and the impact of adjustment for BMI on the smoking-LTL association analyses have not been reported. The present study aims to dissect the direct effect of smoking on LTL and its indirect effect through BMI and to distinguish the mediation and suppression effects of body weight in a study cohort of African American (AA) and white adults from the Bogalusa Heart Study.

## RESULTS

### Participant characteristics

The cohort characteristics are displayed in Table 1. Age had significant sex difference (males>females) in AAs. BMI had significant race differences (AAs>Whites) in females and opposite trends in sex differences in Whites (males>females) versus AAs (males<females). Heavy alcohol use had sex difference in AAs (males>females) and race difference in males (AAs>Whites). Prevalence of smoking was significantly higher in AA males than white males. Although log-transformed pack-years of smoking was used for regression analyses, its original values were presented in Table 1. The mean of pack-years of smoking was significantly greater in white males than in white females and was significantly greater in Whites than in AAs. AAs had significantly longer LTL than Whites.

**Table 1:** Descriptive data of study participants by race and sex.

	**Whites**		**African Americans**		**P for race difference**	
	**Male (n=322)**	**Female (n=407)**	**Male (n=115)**	**Female (n=193)**	**Male**	**Female**
Age (year)	40.9 (6.4)	39.9 (7.2)	41.3 (6.3)	39.6 (7.1)*	0.488	0.619
BMI (kg/m^2^)	30.1 (5.9)	28.8 (7.3)*	30.6 (8.0)	33.0 (9.3)*	0.496	<0.001
Alcohol use, n (%)					<0.001	0.111
No	133 (41.3)	155 (38.1)	43 (37.4)	89 (46.1)*		
Light	131 (40.7)	194 (47.7)	29 (25.2)	75 (38.9)*		
Heavy	58 (18.0)	58 (14.2)	43 (37.4)	29 (15.0)*		
Smokers, n (%)	81 (25.2)	122 (30.0)	47 (40.9)	59 (30.6)	0.002	0.882
Pack-years^a^	16.8 (12.0)	12.8 (9.4)*	10.6 (7.9)	8.2 (6.9)	<0.001	<0.001
LTL (kb)	6.827 (0.663)	6.921 (0.721)	7.274 (0.773)	7.456 (0.732)	<0.001	<0.001

BMI=body mass index; LTL=leukocyte telomere length

a, Pack-years of smoking

*, P<0.05 for sex difference

### The effects of smoking and BMI on LTL

We present results of associations between smoking (yes/no), BMI and LTL in 3 models in Table 2 based on standardized regression coefficient (β). Model 1 shows a significant effect of smoking on LTL without BMI included. The effects of smoking and BMI on LTL were significant in Model 2 when both BMI and smoking were included in the model. The effect size of smoking on LTL (β= -0.072, p=0.012) in Model 2 was greater compared to that (β= -0.061, p=0.034) in Model 1 in terms of their absolute values. Model 3 presents the effect of smoking on BMI (β= -0.143, p<0.001). The results were substantially similar when pack-years of smoking was used in models 4, 5 and 6 in Table 2.

**Table 2:** Standardized regression coefficients of smoking on LTL.

	**Model 1**			**Model 2**		
	β	SE	P	β	SE	P
Age	-0.266	0.029	<0.001	-0.260	0.029	<0.001
AA race	0.310	0.028	<0.001	0.325	0.029	<0.001
Female sex	0.054	0.028	0.058	0.053	0.028	0.063
Alcohol drinking	-0.044	0.028	0.125	-0.052	0.029	0.068
BMI	---	---	---	-0.080	0.029	0.006
Smoking	-0.061	0.029	0.034	-0.072	0.029	0.012
	**Model 4**			**Model 5**		
	β	SE	P	β	SE	P
Age	-0.267	0.028	<0.001	-0.260	0.028	<0.001
AA race	0.307	0.028	<0.001	0.321	0.029	<0.001
Female sex	0.052	0.028	0.069	0.050	0.028	0.077
Alcohol drinking	-0.045	0.028	0.115	-0.054	0.028	0.058
BMI	---	---	---	-0.085	0.029	0.004
Pack-years	-0.082	0.028	0.004	-0.094	0.029	0.001

**Model 3**: Effect of smoking on BMI: β= -0.143, SE=0.030, P<0.001

**Model 6**: Effect of pack-years on BMI: β= -0.150, SE=0.030, P<0.001

β=standardized regression coefficients; SE=standard error; LTL=leukocyte telomere length; AA=African Americans; BMI=body mass index

### Suppression effect of BMI

**Figure 1:**
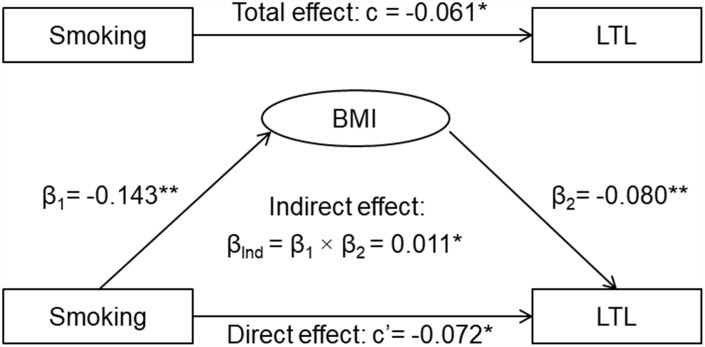
**General third variable model of smoking, BMI and LTL.** β, c and c’ are standardized regression coefficients; c=total effect; c’=direct effect; β_1_=indirect effect 1; β_2_=indirect effect 2; β_Ind_=total indirect effect; BMI=body mass index; LTL=leukocyte telomere length. * P<0.05; ** P<0.01.

**Figure 2:**
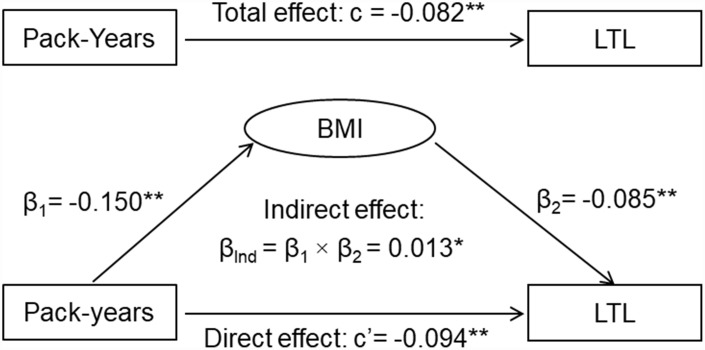
**General third variable model of pack-years of smoking, BMI and LTL.** β, c and c’ are standardized regression coefficients; c=total effect; c’=direct effect; β_1_=indirect effect 1; β_2_=indirect effect 2; β_Ind_=total indirect effect; BMI=body mass index; LTL=leukocyte telomere length. * P<0.05; ** P<0.01.

The results of the third variable model analyses are shown in Figure 1. The overall indirect effect (β_Ind_) of smoking on LTL through BMI was measured as the product of indirect effect 1 and indirect effect 2 (β_Ind_ = β_1_ × β_2_). The overall indirect effect of smoking on LTL through BMI was estimated at β_Ind_ =0.011, p=0.023. The direct effect of smoking on LTL (c’= -0.072, p=0.012) was greater than the total effect (c= -0.061, p=0.034) in terms of their absolute values, indicating that BMI was a suppressor, not a mediator, of the relationship between smoking and LTL. A similar suppression effect by BMI was observed when pack-years of smoking was used (Figure 2).

### The effects of smoking and BMI on LTL by race and sex

The differences in the total, indirect and direct effects of smoking on LTL between race and sex groups were tested for significance in regression interaction models. All the effect parameters did not differ significantly between race and sex groups (Table 3).

**Table 3:** Differences in effects of smoking and BMI on LTL between race and sex groups.

	**Total effect: Smoking→LTL ©^a^**	**Indirect effect 1: Smoking→BMI (β_1_)**	**Indirect effect 2: BMI→LTL (β_2_)**	**Direct effect: Smoking→LTL (c’)^b^**
Whites (n=729)	-0.076 *	-0.136 *	-0.113 *	-0.091 *
African Americans (n=308)	-0.010	-0.141 *	-0.041	-0.016
P for race difference	0.539	0.477	0.104	0.421
Males (n=437)	-0.053	-0.196 *	-0.099 *	-0.072
Females (n=600)	-0.064	-0.104 *	-0.073	-0.072
P for sex difference	0.865	0.244	0.515	0.971

β, c and c’ are standardized regression coefficients.

BMI=body mass index; LTL=leukocyte telomere length

a, BMI was not included in the models for adjustment.

b, BMI was included in the models for adjustment.

*, P<0.05

## DISCUSSION

Our findings suggest that BMI modifies the association between cigarette smoking and LTL by suppressing the effect of smoking, which potentially contributes to the mixed findings reported on the smoking-LTL association in previous studies [13]. Other causes for the inconsistent findings might be suboptimal LTL measurements, small sample size, age range, sex, race, statistical analysis models and the number of covariates included, and often their complex interactions [4–7, 19–21].

A large body of research has focused on the relationship between smoking and LTL shortening. There are, however, considerable inconsistencies in the reported associations. In a systematic review of 84 studies, 33 studies reported shorter LTL with smoking, one study reported longer LTL with smoking, and 50 studies found a lack of significant associations between smoking and LTL despite a trend of shorter LTL with smoking in the majority of these 50 studies [13]. The inconsistencies in the smoking-LTL relationship in previous studies may be due to BMI that is related to both smoking and LTL.

By definition [22], BMI as the third variable is not a confounder for the smoking-LTL association because it is intermediate in the causal path between the predictor and the outcome as shown in Figure 1. In this study, we found that the absolute value of the direct effect of smoking was significantly increased, indicating that the smoking-LTL association would be underestimated or suppressed without adjustment for BMI. In the systematic review [13], among the 26 studies with a sample size ≥1000, five (83.3%) out of 6 studies that had BMI included for adjustment reported significant associations (direct effect), and 12 (60.0%) out of 20 studies that did not have BMI included for adjustment reported nonsignificant associations (total effect). These large-scale studies showed a trend towards the suppression effect of BMI on the smoking-LTL association. The results of the current study provided an explanation on the inconsistency of the smoking-LTL relationship in the literature [13].

The mechanisms of smoking-related LTL shortening through increased inflammation and oxidative stress are well-known. Oxidative stress accelerates telomere erosion during somatic cell replication, and inflammation

increases leukocyte turnover rate [10–12, 23]. Numerous studies have demonstrated that obesity is another important risk factor for telomere shortening [14–16]. Although the inverse association between obesity and cigarette smoking has long been established [17, 18], the mechanisms underlying their joint effect on LTL through changes in inflammation and oxidative stress are not well understood. To the best of our knowledge, the complex relationship between smoking, BMI and LTL has not been dissected in earlier population studies.

It is widely believed that there are considerable AA-white and sex differences in LTL (AAs>whites and females>males) [24, 25]. In this study, we observed a significant race effect and borderline sex effect on LTL. In a previous cross-sectional analysis of LTL in a combined sample of two studies, LTL was longer in AAs and females than in Whites and males, respectively, in the NHLBI Family Heart Study participants (age range, 30-93 years), but the sex difference in LTL was not significant in the Bogalusa Heart Study participants (age range, 19-37 years) [24]. A potential explanation is that the size of difference in sex effect on LTL may be related to age periods.

Despite the marked AA-white difference in LTL observed in this study, the total, direct and indirect effects of smoking on LTL did not differ significantly between AAs and Whites. These findings are consistent with the Women’s Health Initiative Study showing that the association between smoking and LTL was significant in both AA and white women [26]. Sex-specific associations between cigarette smoking and saliva telomere length were seen among 5,624 older adults in the Health and Retirement Study [4]. In the present study, however, the total, indirect and direct effects of smoking on LTL did not show significant differences between males and females.

This observational population study has certain limitations. First, this study cohort was too young (mean age=40.3 years, age range=26.2-50.1 years) to have cardiovascular morbidity and mortality outcomes. Therefore, we did not have sufficient data to examine the effects of LTL on cardiovascular events. Second, physical activity data were not available in this cohort, and its impact on the smoking-LTL association could not be examined. Third, chronic inflammation and oxidative stress data were available only in part of the study cohort; further research is needed to investigate their mediation or suppression effects on the smoking-LTL association.

We conclude that body weight, the third external variable in the causal path from smoking to LTL shortening, exerts a suppression effect, not a mediation effect, on the smoking-LTL association. The findings of the current study have biological and practical implications by providing better understanding of the smoking-LTL relationship and demonstrating how BMI influences the estimation of the effect of cigarette smoking on LTL. The changes in chronic inflammation and oxidative stress among obese smokers and lean non-smokers may underlie the joint effect of cigarette smoking and obesity on LTL, which should be examined in future studies.

## MATERIALS AND METHODS

### Study cohort

The Bogalusa Heart Study focuses on the natural history of cardiovascular disease since childhood in a semirural, biracial (65% white and 35% AA) community of Bogalusa, Louisiana [27]. The current study cohort consisted of 729 white and 308 AA adults (42.1% males; mean age=40.3 years, age range=26.2~50.1 years) with data available on LTL, BMI, cigarette smoking and alcohol drinking history.

All participants in this study gave informed consent. Study protocols were approved by the Institutional Review Board of the Tulane University Health Sciences Center.

### Measurements

Height and weight were measured in duplicate, and the mean values were used for analysis. BMI was calculated as weight in kilograms divided by height in meters squared. Information on smoking status and duration and alcohol drinking was obtained in a questionnaire survey. Current smokers were defined as smoking at least one cigarette per day during the past 12 months. Light and heavy drinkers were defined as drinking alcohol 1-2 times and ≥3 times per week, respectively, during the past 12 months. LTL was measured by Southern blots of the terminal restriction fragments [28].

### Statistical methods

Analyses of covariance and Chi-square tests were performed to test differences in continuous and categorical study variables, respectively, between race and sex groups. Pack-years of smoking was log-transformed for analysis. The effect of smoking, log-transformed pack-years of smoking and BMI on LTL was examined in multivariable linear regression analysis models, adjusted for age, race, sex and alcohol drinking. The significance of differences in the regression coefficients between race and sex groups was tested in interaction regression models by including smoking-race or smoking-sex interaction terms.

Although there are distinct conceptual differences among mediation, confounding and suppression, these three types of effects share statistical similarities in estimation of the parameters. Each effect is quantified by measuring the change in the relationship between independent and dependent variables after adding a third variable to the analysis models [19]. The general third variable model [19], specifically the causal mediation analysis model [29], was constructed to distinguish the mediation and suppression effects of BMI on the association between smoking and LTL. Smoking was the predictor variable (X), BMI the mediator (M) or suppressor, and LTL the dependent variable (Y). The third variable effect analyses were performed using multivariable linear regression models, adjusted for age, race, sex and alcohol drinking. In general, there are four steps for the analyses: 1) showing that smoking determines LTL (Model Y = c X) where c is total effect; 2) showing that smoking affects BMI (Model M = β_1_ X) where β_1_ is indirect effect 1; 3) showing that BMI determines LTL controlling for smoking (Model Y = β_2_ M + c’ X) where β_2_ is indirect effect 2, and c’ is direct effect; 4) determining mediation effect and suppression effect by comparing the magnitude (the absolute values) of the total effect © and direct effect (c’). A mediation effect is suggested if c>c’; a suppression effect is suggested if c<c’ [19].

## References

[1] Blackburn EH. Switching and signaling at the telomere. Cell. 2001; 106:661–73. 10.1016/S0092-8674(01)00492-5 11572773

[2] Edo MD, Andrés V. Aging, telomeres, and atherosclerosis. Cardiovasc Res. 2005; 66:213–21. 10.1016/j.cardiores.2004.09.007 15820190

[3] Fyhrquist F, Saijonmaa O. Telomere length and cardiovascular aging. Ann Med. 2012 (Suppl 1); 44:S138–42. 10.3109/07853890.2012.660497 22713142

[4] Zhang C, Lauderdale DS, Pierce BL. Sex-specific and time-varying associations between cigarette smoking and telomere length among older adults. Am J Epidemiol. 2016; 184:922–32. 10.1093/aje/kww102 27856448 PMC5161083

[5] Valdes AM, Andrew T, Gardner JP, Kimura M, Oelsner E, Cherkas LF, Aviv A, Spector TD. Obesity, cigarette smoking, and telomere length in women. Lancet. 2005; 366:662–64. 10.1016/S0140-6736(05)66630-5 16112303

[6] Starkweather AR, Alhaeeri AA, Montpetit A, Brumelle J, Filler K, Montpetit M, Mohanraj L, Lyon DE, Jackson-Cook CK. An integrative review of factors associated with telomere length and implications for biobehavioral research. Nurs Res. 2014; 63:36–50. 10.1097/NNR.0000000000000009 24335912 PMC4112289

[7] Latifovic L, Peacock SD, Massey TE, King WD. The influence of alcohol consumption, cigarette smoking, and physical activity on leukocyte telomere length. Cancer Epidemiol Biomarkers Prev. 2016; 25:374–80. 10.1158/1055-9965.EPI-14-1364 26656293

[8] Yanbaeva DG, Dentener MA, Creutzberg EC, Wesseling G, Wouters EF. Systemic effects of smoking. Chest. 2007; 131:1557–66. 10.1378/chest.06-2179 17494805

[9] van der Vaart H, Postma DS, Timens W, ten Hacken NH. Acute effects of cigarette smoke on inflammation and oxidative stress: a review. Thorax. 2004; 59:713–21. 10.1136/thx.2003.012468 15282395 PMC1747102

[10] von Zglinicki T. Oxidative stress shortens telomeres. Trends Biochem Sci. 2002; 27:339–44. 10.1016/S0968-0004(02)02110-2 12114022

[11] Aviv A. Telomeres and human aging: facts and fibs. Sci SAGE KE. 2004; 2004:pe43. 10.1126/sageke.2004.51.pe43 15618136

[12] O’Donovan A, Pantell MS, Puterman E, Dhabhar FS, Blackburn EH, Yaffe K, Cawthon RM, Opresko PL, Hsueh WC, Satterfield S, Newman AB, Ayonayon HN, Rubin SM, et al, and Health Aging and Body Composition Study. Cumulative inflammatory load is associated with short leukocyte telomere length in the Health, Aging and Body Composition Study. PLoS One. 2011; 6:e19687. 10.1371/journal.pone.0019687 21602933 PMC3094351

[13] Astuti Y, Wardhana A, Watkins J, Wulaningsih W, and PILAR Research Network. Cigarette smoking and telomere length: A systematic review of 84 studies and meta-analysis. Environ Res. 2017; 158:480–89. 10.1016/j.envres.2017.06.038 28704792 PMC5562268

[14] Gielen M, Hageman GJ, Antoniou EE, Nordfjall K, Mangino M, Balasubramanyam M, de Meyer T, Hendricks AE, Giltay EJ, Hunt SC, Nettleton JA, Salpea KD, Diaz VA, et al, and TELOMAAS group. Body mass index is negatively associated with telomere length: a collaborative cross-sectional meta-analysis of 87 observational studies. Am J Clin Nutr. 2018; 108:453–75. 10.1093/ajcn/nqy107 30535086 PMC6454526

[15] Müezzinler A, Zaineddin AK, Brenner H. Body mass index and leukocyte telomere length in adults: a systematic review and meta-analysis. Obes Rev. 2014; 15:192–201. 10.1111/obr.12126 24165286

[16] Mundstock E, Sarria EE, Zatti H, Mattos Louzada F, Kich Grun L, Herbert Jones M, Guma FT, Mazzola In Memoriam J, Epifanio M, Stein RT, Barbé-Tuana FM, Mattiello R. Effect of obesity on telomere length: systematic review and meta-analysis. Obesity (Silver Spring). 2015; 23:2165–74. 10.1002/oby.21183 26407932

[17] Molarius A, Seidell JC, Kuulasmaa K, Dobson AJ, Sans S. Smoking and relative body weight: an international perspective from the WHO MONICA Project. J Epidemiol Community Health. 1997; 51:252–60. 10.1136/jech.51.3.252 9229053 PMC1060469

[18] Lv J, Chen W, Sun D, Li S, Millwood IY, Smith M, Guo Y, Bian Z, Yu C, Zhou H, Tan Y, Chen J, Chen Z, Li L, and China Kadoorie Biobank collaborative group. Gender-specific association between tobacco smoking and central obesity among 0.5 million Chinese people: the China Kadoorie Biobank Study. PLoS One. 2015; 10:e0124586. 10.1371/journal.pone.0124586 25897789 PMC4405570

[19] MacKinnon DP, Krull JL, Lockwood CM. Equivalence of the mediation, confounding and suppression effect. Prev Sci. 2000; 1:173–81. 10.1023/A:1026595011371 11523746 PMC2819361

[20] Verhulst S, Susser E, Factor-Litvak PR, Simons MJ, Benetos A, Steenstrup T, Kark JD, Aviv A. Commentary: the reliability of telomere length measurements. Int J Epidemiol. 2015; 44:1683–86. 10.1093/ije/dyv166 26403812 PMC4681112

[21] Aviv A, Hunt SC, Lin J, Cao X, Kimura M, Blackburn E. Impartial comparative analysis of measurement of leukocyte telomere length/DNA content by Southern blots and qPCR. Nucleic Acids Res. 2011; 39:e134. 10.1093/nar/gkr634 21824912 PMC3203599

[22] Weinberg CR. Toward a clearer definition of confounding. Am J Epidemiol. 1993; 137:1–8. 10.1093/oxfordjournals.aje.a116591 8434568

[23] Forsyth NR, Evans AP, Shay JW, Wright WE. Developmental differences in the immortalization of lung fibroblasts by telomerase. Aging Cell. 2003; 2:235–43. 10.1046/j.1474-9728.2003.00057.x 14570231

[24] Hunt SC, Chen W, Gardner JP, Kimura M, Srinivasan SR, Eckfeldt JH, Berenson GS, Aviv A. Leukocyte telomeres are longer in African Americans than in whites: the National Heart, Lung, and Blood Institute Family Heart Study and the Bogalusa Heart Study. Aging Cell. 2008; 7:451–58. 10.1111/j.1474-9726.2008.00397.x 18462274 PMC2810865

[25] Gardner M, Bann D, Wiley L, Cooper R, Hardy R, Nitsch D, Martin-Ruiz C, Shiels P, Sayer AA, Barbieri M, Bekaert S, Bischoff C, Brooks-Wilson A, et al, and Halcyon study team. Gender and telomere length: systematic review and meta-analysis. Exp Gerontol. 2014; 51:15–27. 10.1016/j.exger.2013.12.004 24365661 PMC4523138

[26] Carty CL, Kooperberg C, Liu J, Herndon M, Assimes T, Hou L, Kroenke CH, LaCroix AZ, Kimura M, Aviv A, Reiner AP. Leukocyte telomere length and risks of incident coronary heart disease and mortality in a racially diverse population of postmenopausal women. Arterioscler Thromb Vasc Biol. 2015; 35:2225–31. 10.1161/ATVBAHA.115.305838 26249011 PMC4713196

[27] Berenson GS, Wattigney WA, Bao W, Srinivasan SR, Radhakrishnamurthy B. Rationale to study the early natural history of heart disease: the Bogalusa Heart Study. Am J Med Sci. 1995 (Suppl 1); 310:S22–28. 10.1097/00000441-199512000-00005 7503119

[28] Kimura M, Stone RC, Hunt SC, Skurnick J, Lu X, Cao X, Harley CB, Aviv A. Measurement of telomere length by the Southern blot analysis of terminal restriction fragment lengths. Nat Protoc. 2010; 5:1596–607. 10.1038/nprot.2010.124 21085125

[29] Sobel ME. Asymptotic confidence intervals for indirect effects in structural equation models. Sociol Methodol. 1982; 13:290–312. 10.2307/270723

